# Endothelial dysfunction markers in cervical cancer and their influence on patient outcome

**DOI:** 10.1007/s11033-026-11448-z

**Published:** 2026-01-13

**Authors:** Juliane Raeck, José Brito da Silva, Luísa Carvalho, Lurdes Salgado, Deolinda Pereira, Beatriz Vieira Neto, Valéria Tavares, Inês Guerra de Melo, Rui Medeiros

**Affiliations:** 1 Molecular Oncology and Viral Pathology Group, Research Center of IPO Porto (CI-IPOP)/CI-IPOP@RISE (Health Research Network), Portuguese Oncology Institute of Porto (IPO Porto)/Pathology and Laboratory Medicine Department/Clinical Pathology/Porto Comprehensive Cancer Center Raquel Seruca (Porto.CCC), Porto, 4200-072 Portugal; 2 Oncology Department, Portuguese Institute of Oncology of Porto (IPO Porto), Porto, 4200-072 Portugal; 3 External Radiotherapy Department, Portuguese Institute of Oncology of Porto (IPO Porto), Porto, 4200-072 Portugal; 4 Research Department, Portuguese League Against Cancer – Northern Branch (LPCC- NRN), Porto, 4200-172 Portugal; 5 Abel Salazar Institute for the Biomedical Sciences (ICBAS), University of Porto, Porto, 4050-313 Portugal; 6 Faculty of Health Sciences, CEBIMED, Fernando Pessoa University, Porto, 4200-150 Portugal

**Keywords:** Uterine cervical neoplasms, Venous thromboembolism, Nitric oxide synthase type III, Genetic markers, Prognosis

## Abstract

**Background:**

Cervical cancer (CC) is a major cause of cancer-related mortality worldwide. Among CC patients, venous thromboembolism (VTE) represents the second leading cause of death, surpassed only by the malignancy itself. This life-threatening condition is characterised by blood stasis, heightened tendency for blood clotting (blood hypercoagulability), and endothelial dysfunction (ED). Single-nucleotide polymorphisms (SNPs) in ED-associated genes are believed to influence an individual’s susceptibility to VTE. Furthermore, these genetic variants may impact treatment response and long-term CC patient outcomes, given the close interaction between cancer and thrombosis.

**Methods and results:**

In this study, the implications of four ED-related SNPs were analysed in a cohort of 379 CC patients. The SNP *NOS3* rs2070744 was significantly associated with the 10-year overall survival (OS) of young patients (≤ 49 years). In addition, this SNP was identified as a predictor of mortality risk in this subgroup, independent of CC stage (< IIB vs. ≥ IIB) and VTE status (yes vs. no) (CC vs. CT/TT; hazard ratio (HR) = 1.90, *p* = 0.025). Incorporating *NOS3* rs2070744 into a predictive clinical model increased prognostic precision regarding patient survival by 15% compared to cancer stage alone. For the remaining SNPs, *NOS3* rs1799983, *vWF* rs1063856 and *SELP* rs6136, no significant association with OS was detected (log-rank test, *p* > 0.05).

**Conclusion:**

These results underscore the role of *NOS3* rs2070744 in CC patients and highlight the potential of integrating genetic markers into prognostic models to support personalised treatment strategies for these patients.

## Introduction

Cervical cancer (CC) is the most common gynaecological malignancy and a leading cause of cancer-related mortality among women, particularly in low- and middle-income countries. It is primarily caused by persistent infection with high-risk human papillomavirus (HPV) types, most notably HPV 16 and 18 [1]. Although vaccination against HPV (primary prevention) effectively prevents the majority of new CC cases, access remains inconsistent across countries [2]. Moreover, as the vaccine is most effective when administered before sexual debut, many adult women remain unvaccinated and susceptible to HPV infection [3]. Consequently, approximately 600,000 new cases of CC are still diagnosed each year globally. In addition to primary prevention by vaccination, regular screening methods to detect CC in early stages (secondary prevention) are of critical importance for improving patient survival: if being treated in early stages (localised cancer), patients have a 5-year survival rate of 91%, compared to a rate of only 19% if the tumour has already spread to distant organs [1, 4].

The poor prognosis observed at advanced cancer stages is attributed not only to more aggressive tumour characteristics, including the induction of angiogenesis and metastasis, but also to a four- to nine-fold increased risk of developing venous thromboembolism (VTE) compared to the general population [5, 6]. Cancer cells actively promote venous thrombogenesis by expressing procoagulant and proangiogenic factors, such as tissue factor (TF) and vascular endothelial growth factor (VEGF), respectively [5, 7, 8]. In addition, conventional treatment approaches like surgery, chemotherapy and radiotherapy significantly increase the chance of thrombotic events. Similarly, emerging treatment strategies, including molecular targeted therapies and immunotherapy, may also disrupt vascular homeostasis and promote thrombus formation [8–10]. Considering the well-established prevalence of VTE across various malignancies, current research efforts should focus not only on advancing cancer treatment strategies but also on better understanding the mechanisms behind VTE onset and developing strategies to prevent this life-threatening complication.

The development of VTE is classically described by the Virchow triad, which includes blood stasis, endothelial dysfunction (ED) and blood hypercoagulability [11, 12]. In cancer patients, ED can result from inflammatory cytokines, tumour-derived proangiogenic and procoagulant factors and oxidative stress in the tumour microenvironment, collectively transforming the endothelium into a prothrombotic and proinflammatory surface that facilitates both VTE and tumour progression [13, 14]. Particularly, the imbalance between endothelium-derived relaxing factors (EDRFs) and constricting factors (EDCFs) favours vasoconstriction and thrombogenesis [12]. The principal EDRF, nitric oxide (NO), is synthesised by endothelial nitric oxide synthase (NOS3), and is considered one of the key factors in maintaining vascular tone, inhibiting platelet aggregation, and modulating inflammation. Consequently, genetic variants in the *NOS3* gene and other ED-related genes may further influence an individual’s susceptibility to VTE, and can potentially influence cancer patient outcomes and treatment success [12, 13, 15]. In this context, the current study explores the prognostic significance of single-nucleotide polymorphisms (SNPs) in genes involved in vascular biology in a cohort of CC patients. The objective of this study is to clarify the potential role of inherited genetic variation in ED-related pathways in contributing to differences in VTE development and CC patient survival, potentially offering novel prognostic markers and/or therapeutic targets for personalised interventions.

## Materials and methods

### Study cohort

The cohort of this retrospective study comprised a total of 379 women of European ancestry diagnosed cytologically and histologically with CC, who were admitted for first-line treatment between February 2002 and October 2009, and again between May 2017 and October 2021 at the Clinic of Gynaecology at the Portuguese Institute of Oncology Porto (IPO Porto). The inclusion criteria required patients to have tumours classified as stage IB2 to IVA according to the International Federation of Gynaecology and Obstetrics (FIGO) and to have received concurrent chemoradiotherapy (CCRT) as first-line treatment, consisting of weekly cisplatin (40 mg/m^2^) administration followed by brachytherapy when clinically indicated. Patients were excluded from the study if they were under 18 years old, had undergone surgery within six months before CCRT, were referred to IPO Porto only for a second opinion, or if their biological samples were absent in the institutional biobank. Written consent, in accordance with the principles of the Helsinki Declaration, was obtained from each patient. The study was previously approved by the ethics committee at IPOP (CES IPO: 287 A/014).

The mean time of follow-up was 155.3 ± 7.6 months. Demographic and clinicopathological information of the patients (Table 1) was obtained by revising medical records. Patients were divided into two subgroups based on age at the time of CC diagnosis, using the median age as a cutoff [≤ 49 versus (vs.) > 49 years]. Additionally, patients were categorised according to their VTE status (with vs. without clinical evidence of VTE). Notably, routine VTE screening is not incorporated into the hospital’s standard diagnostic protocol. Consequently, only cases with symptomatic VTE events could be considered. A third classification was tumour histology, which categorises cases into squamous cell carcinoma (SCC), adenocarcinoma (ADC) and other less common subtypes.

**Table 1:** Clinicopathological characteristics of CC patients according to age at tumour diagnosis (*N* = 379)

Variable	Age at CC diagnosis	
	≤ 49 years* (*N* = 209, 55.1%)	> 49 years* (*N* = 170, 44.9%)
*VTE status* (*N* = 378)		
With VTE	12 (5.7)	9 (5.3)
Without clinical evidence of VTE	197 (94.3)	160 (94.1)
Missing data	–	1 (0.6)
*FIGO stage* (*N* = 372)		
I - IIA	39 (18.7)	15 (8.8)
IIB - IV	166 (79.4)	152 (89.4)
Missing data	4 (1.9)	3 (1.8)
*Cancer histology* (*N* = 254)		
Adenocarcinoma and others	26 (12.4)	16 (9.4)
Squamous cell carcinoma	117 (56.0)	95 (55.9)
Missing data	66 (31.6)	59 (34.7)

*Cut-off defined based on the median value of age. Abbreviations: CC, cervical cancer; FIGO, International Federation of Gynaecology and Obstetrics; N, number of patients; VTE, venous thromboembolism.

### Sample collection and genomic DNA isolation

Before treatment initiation, peripheral venous blood samples were obtained from each patient into EDTA-containing tubes, using a standard phlebotomy technique.

Genomic DNA was isolated from the blood samples using the QUIamp DNA Blood Mini Kit (Cat. No. 51106, Qiagen, Hilden, Germany), following the manufacturer’s protocol. The concentration and purity of the DNA samples were verified using a NanoDrop Lite Spectrophotometer (Thermo Fisher Scientific^®^, Waltham, MA, USA) before storage at -20 °C until subsequent use.

### Selection of germline variations

The selection of SNPs to be evaluated in the study was based on: (1) their involvement in ED and venous thrombogenesis pathways, (2) their known associations with cardiovascular and metabolic disorders, and malignancies, supporting their relevance as potential biomarkers and (3) the availability of Taqman genotyping assays. According to these criteria, the following SNPs were selected: *NOS3* rs1799983 and rs2070744, *von Willebrand factor* (*vWF*) rs1063856, and *P-Selectin* (*SELP*) rs6136.

### Polymorphism genotyping

SNP genotyping was performed using the TaqMan^®^ Allelic Discrimination method in a StepOne Plus Real-time PCR system (Applied Biosystems^®^, Foster City, CA, USA). Each reaction comprised a mixture of 6.0 µL, including 2.5 µL of TaqPath™ ProAmp™ Master Mix (1×), 2.375 µL of sterile water, 0.125 µL of TaqMan^®^ Genotyping Assay Mix (C___3219460_20 for *NOS3* rs1799983, C__15903863_10 for *NOS3* rs2070744, C___3288406_30 for *VWF* rs1063856 and C__11975277_20 for *SELP* rs6136), and 1.0 µL of genomic DNA. To ensure reliable amplification, negative controls (without DNA) were included in each reaction [16].

The DNA amplification followed a three-step thermal cycling protocol, initiated by an enzyme activation step at 95 °C for 10 min, followed by 45 cycles of denaturation at 95 °C for 15 s, and primer annealing/extension at 60 °C for 1 min. The data analysis was conducted by using StepOne Software (version 2.3, Applied Biosystems^®^). In order to verify the accuracy of the SNP genotyping, a double-sampling strategy was applied to at least 10% of randomly selected samples, with a genotyping accuracy that exceeded 99%.

The genotyping results were independently evaluated by three researchers blinded to patient clinical data in order to avoid detection bias.

### Statistical analysis

Descriptive statistics were used to summarise the clinical and demographic characteristics of the study population. Due to the large cohort size, data distribution was assessed using the Kolmogorov-Smirnov test. The genotype distributions of each SNP were compared to those reported in the Iberian population. Deviations from Hardy-Weinberg equilibrium (HWE) were evaluated using the chi-square (χ^2^) test, as well as associations between SNP genotypes and clinical or demographic characteristics, including VTE status (with vs. without clinical evidence of VTE), FIGO stage (< IIB vs. ≥ IIB), and histological subtype (SCC vs. ADC and others).

10-year overall survival (OS) was defined as the time from CC diagnosis to death or last follow-up. Survival curves were estimated using the Kaplan-Meier method. Differences in survival distributions between genotypes were analysed using the log-rank test or the Tarone-Ware test. The most appropriate genetic model (dominant or recessive) for each SNP was selected based on the results of the additive genetic analysis. A stratified analysis by age (≤ 49 vs. > 49 years) was conducted for SNPs demonstrating at least marginal associations in the overall cohort (*p* < 0.1), concerning VTE incidence and OS.

Cox regression models were utilised in order to estimate hazard ratios (HRs) and 95% confidence intervals (CIs) regarding the development of VTE and patient death. Both univariate and multivariate approaches were applied for the relevant SNPs to account for potential confounding effects. To evaluate the stability and reliability of the observed HRs, bootstrap resampling (1,000 iterations) was performed.

The predictive value of prognostic models combining clinical parameters with SNPs was analysed using multivariate Cox regression analysis to estimate the associations with patient mortality. Only clinical factors identified as prognostic in the univariate Cox analysis were included. Model discrimination was measured using the calculated concordance index (c-index), with higher values indicating superior predictive performance. Internal validation was performed via Bootstrap resampling (1,000 iterations).

All statistical analyses were conducted using the IBM SPSS Statistics for Windows (version 29, IBM Corp., Armonk, NY, USA). All statistical tests were two-sided, with a significance level set at *p* < 0.05.

## Results

### Impact of clinical parameters on CC prognosis

In the study, 6.1% of the patients (23 out of 379) presented with symptomatic VTE. Those affected by the condition had a lower 10-year OS compared to those without clinical evidence of VTE (mean 10-year OS of 63.2 ± 11.3 months and 91.7 ± 2.4 months, respectively; log-rank test, *p* = 0.008) [17].

Staging according to the FIGO system was documented for 98.2% of the patients (372 out of 379). 10-year OS significantly decreased with more advanced stages, when classifying stages as I, II, III and IV, with the mean 10-year OS being 105.6 ± 6.2 months, 92.5 ± 2.8 months, 80.8 ± 5.8 months and 62.9 ± 14.1 months, respectively (Tarone-Ware test, *p* = 0.003). Additionally, when distinguishing between early (I and II) and advanced stages (III and IV), a significant decrease in 10-year OS was observed in the latter group (mean 10-year OS of 94.1 ± 2.6 months and 78.2 ± 5.4 months, respectively; log-rank test, *p* = 0.004). Using an alternative classification system, wherein early stages were defined as stages < IIB and advanced stages as ≥ IIB, advanced disease was once more associated with lower OS (mean 10-year OS of 104.8 ± 4.8 months and 87.8 ± 2.7 months, respectively; Tarone-Ware test, *p* = 0.02).

Data on CC histology were available for 67% of the patients (254 out of 279). No significant association was detected between CC histology and patient 10-year OS (log-rank test, *p* > 0.05). Likewise, no significant association was detected between patient age at cancer diagnosis (≤ 49 vs. >49 years) and 10-year OS (log-rank test, *p* > 0.05).

### Genotype distribution of the ED-associated SNPs

The genotype frequencies for each SNP are represented in Table 2. The HWE analysis confirmed that all germline variants, except for *NOS3* rs1799983, adhere to the expected genotype frequencies (χ^2^ test, *p* > 0.05).

**Table 2:** Genotype distribution of ED-related SNPs in the study cohort (*N* = 379)

SNP	MAFi^1^ (MA)	Genotype	Genotype frequency in the study (%)	Total* N (%)	MaFs (MA)
*NOS3* rs1799983	38.3% (T)	GG	160 (44.8)	357 (100)	35.4% (T)
		GT	141 (39.5)		
		TT	56 (15.7)		
*NOS3* rs2070744	49.5% (C)	TT	117 (31.4)	373 (100)	44.4% (C)
		CT	181 (48.5)		
		CC	75 (20.1)		
*vWF* rs1063856	34.1% (C)	TT	157 (42.1)	373 (100)	35.8% (C)
		CT	165 (44.2)		
		CC	51 (13.7)		
*SELP* rs6136	8.9% (G)	TT	313 (84.8)	369 (100)	8.0% (G)
		GT	53 (14.4)		
		GG	3 (0.8)		

*There were some cases with failed genotyping. ^1^According to the Ensembl database (https://www.ensembl.org/index.html; last accessed on 01 May 2025). Abbreviations: ED, endothelial dysfunction; MA, minor allele; MAFi, minor allele frequency in the Iberian population; MAFs, minor allele frequency in the study cohort; N, number of patients; SNP, Single-nucleotide polymorphism

### ED-related SNPs: associations with VTE development

Among the evaluated SNPs, only *NOS3* rs1799983 demonstrated a significant association with VTE occurrence (χ^2^ test, *p* = 0.023). Patients with the GG genotype exhibited a VTE incidence of 10.1% compared to only 3.6% among carriers of the T allele. This corresponds to an approximately three-fold increased risk of venous thrombotic events within the GG genotype group (odds ratio (OR) = 3.00, 95% CI = 1.22–7.58). Notably, *NOS3* rs1799983 did not adhere to the expected genotype frequencies in the cohort (χ^2^ test, *p* > 0.05).

No additional significant associations with VTE status were observed in the overall cohort or within the subgroups (χ^2^ test, *p* > 0.05).

### ED-related snps: associations with patient OS

**Fig. 1:**
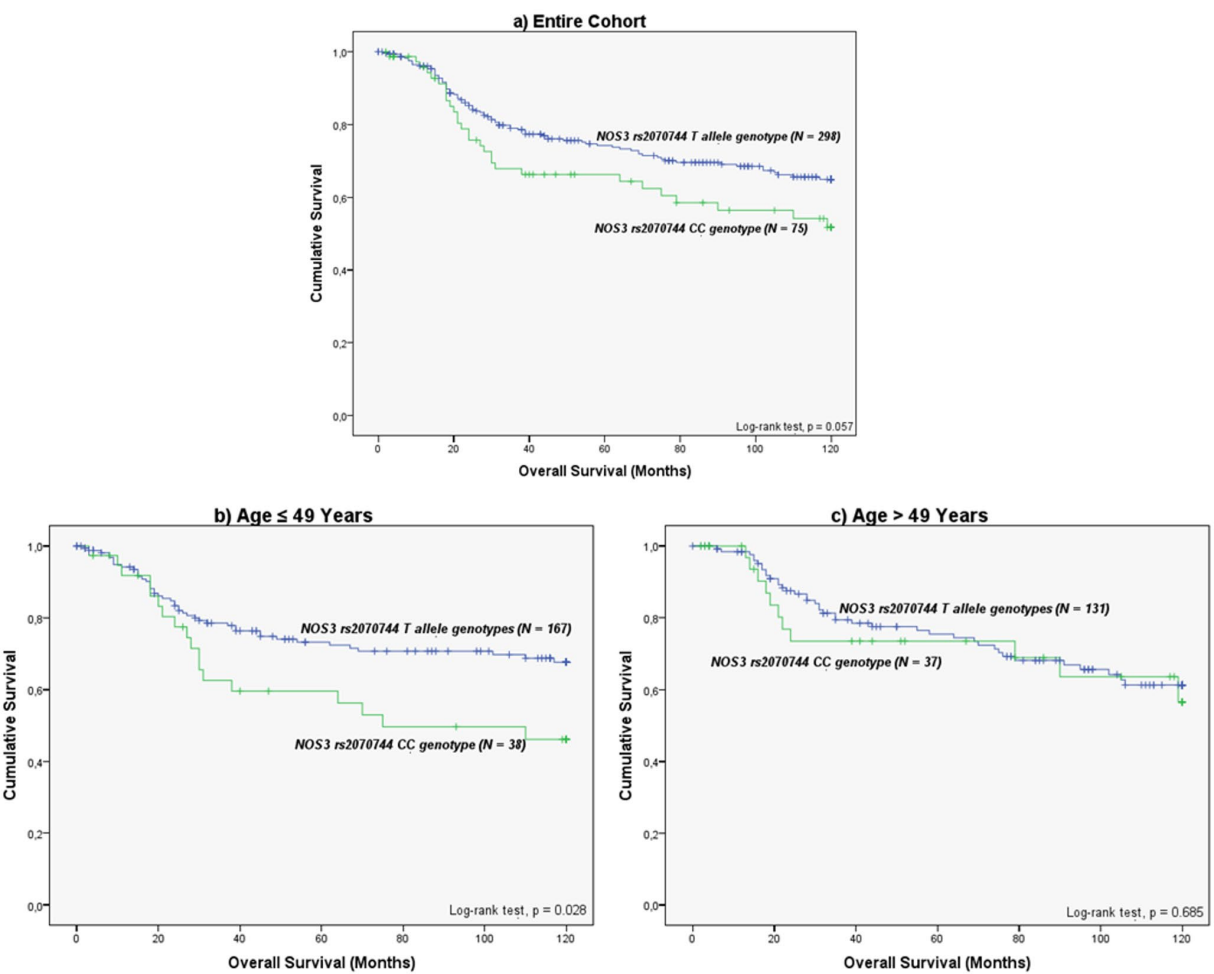
10-year overall survival (OS) by Kaplan-Meier and log-rank test for CC patients, according to *NOS3* rs2070744 (*N* = 379): **(a)** In the entire cohort, while only marginally significant, women with the CC genotype presented a lower OS compared to T allele carriers (10-year OS of 82.0 ± 5.7 months and 92.3 ± 2.6 months, respectively; log-rank test, *p* = 0.057). **(b)** The negative effect of the SNP CC genotype was verified when focusing on younger women (≤ 49 years) (10-year OS of 75.2 ± 7.9 months and 92.3 ± 3.6 months, respectively; log-rank test, *p* = 0.028). **(c)** In contrast, no significant impact of the SNP was observed in the subgroup of older women (10-year OS of 89.5 ± 8.1 months and 92.2 ± 3.8 months, respectively; log-rank test, *p* = 0.068)

In the overall cohort of CC patients, only a marginally significant association was identified, which concerns the SNP *NOS3* rs2070744. Specifically, patients with the CC genotype had a reduced 10-year OS compared to those with the T allele (10-year OS of 82.0 ± 5.7 months and 92.3 ± 2.6 months, respectively; log-rank test, *p* = 0.057; Fig. 1a), suggesting a potential protective effect of the T allele.

Upon stratification by patient age at tumour diagnosis, a significant association was identified among the younger women (≤ 49 years). Namely, individuals with the *NOS3* rs2070744 CC genotype exhibited a significantly lower 10-year OS compared to those with the TT or CT genotypes (10-year OS of 75.2 ± 7.9 months and 92.3 ± 3.6 months, respectively; log-rank test, *p* = 0.028; Fig. 1b). In contrast, in the older cohort of CC patients (> 49 years), no significant association between the SNP and OS was detected (log-rank test, *p* = 0.685; Fig. 1c).

Multivariate Cox regression analysis indicated an almost doubled mortality risk associated with the *NOS3* rs2070744 CC genotype in the subgroup of young women, adjusting for the covariates FIGO stage and VTE status (CC vs. CT/TT; adjusted HR = 1.90, 95% CI = 1.09–3.33, *p* = 0.025). This result remained robust following bootstrap resampling with 1,000 iterations (*p* = 0.008).

The remaining three SNPs (*NOS3* rs1799983, *vWF* rs1063856 and *SELP* rs6136) demonstrated no significant associations with OS in the overall cohort or within the subgroups (log-rank test or Tarone-Ware test, *p* > 0.05).

### ED-related snps: predictive models

Based on identified predictors of CC patient death - *NOS3* rs2070744, FIGO stage and VTE status - multivariate Cox analyses were conducted, focusing on young women (≤ 49 years). Model performance was evaluated using the c-index, where a value of 1 indicates perfect concordance (**Table **3). Model 4, which combines FIGO stages and VTE status with the SNP *NOS3* rs2070744, achieved the highest predictive value (c-index = 0.685). This model increased the predictive ability by 15% compared to Model 1 (FIGO staging alone).

**Table 3:** Prognostic models for 10-year OS in young women at CC diagnosis (≤ 49 years), according to different genetic and clinical parameters

Prognostic models	aHR	95% CI	*p*-Value	*p* Bootstrap	c-index
*Model 1*					
FIGO stage (< IIB^1^ vs. ≥ IIB)	4.4	1.6–12.11	0.004	0.005	0.595
*Model 2*					
FIGO stage (< IIB^1^ vs. ≥ IIB)	3.91	1.41–10.83	0.009	0.010	0.638
VTE status (with vs. without VTE^1^)	4.42	2.14–9.14	< 0.001	0.001	
*Model 3*					
FIGO stage (< IIB^1^ vs. ≥ IIB)	4.32	1.57–11.93	0.005	0.007	0.647
*NOS3* rs2070744 (CC vs. CT/TT^1^)	1.84	1.05–3.23	0.033	0.027	
*Model 4*					
FIGO stage (< IIB^1^ vs. ≥ IIB)	3.82	1.38–10.61	0.010	0.019	0.685
VTE status (with vs. without VTE^1^)	4.71	2.27–9.79	< 0.001	0.001	
*NOS3* rs2070744 (CC vs. CT/TT^1^)	1.90	1.09–3.33	0.025	0.013	

aHR, adjusted hazard ratio; CC, cervical cancer; CI, confidence interval; OS, overall survival; VTE, venous thromboembolism. ^1^ Reference group

## Discussion

VTE is a well-recognised and serious complication in cancer patients, yet its precise incidence and prognostic implications remain underexplored in CC [17–19]. This prothrombotic state is not only a byproduct of cancer, it also actively contributes to tumour progression, significantly increasing patient mortality [11, 14]. In this study, 6.1% of the CC patients (23 out of 379) presented symptomatic VTE, a rate comparable to previous clinical investigations, reporting that between 3% and 34% of CC patients under CCRT exhibit VTE [20, 21]. Importantly, symptomatic VTE was associated with a significantly reduced 10-year OS, with affected CC patients showing a survival of 63.2 ± 11.3 months compared to 91.7 ± 2.4 months in non-VTE patients (log-rank test, *p* = 0.008). This aligns with previous reports of our research group, where the occurrence of VTE in CC patients leads to a nearly three-fold decrease in 10-year OS [17]. This further supports the role of VTE as a negative prognostic factor in CC, which can be explained by the bidirectional relationship between cancer and VTE: tumour cells favour a prothrombotic environment, which in turn enhances tumour growth, metastasis and angiogenesis [8]. Consequently, VTE development is an indicator of aggressive tumour behaviour.

Among other factors, ED plays a critical role in venous thrombogenesis. ED is defined as an impairment of endothelial cell function, which alters vascular tone, inflammatory responses, and coagulation pathways, shifting the properties of endothelial cells from anticoagulant to procoagulant, facilitating both VTE development and tumour progression [12]. As such, genetic markers influencing ED may offer further insights into VTE susceptibility and cervical tumorigenesis. Given their dual relevance, SNPs in ED-related genes have been increasingly recognised as potential prognostic indicators in oncology, regardless of VTE status [18]. Taken together, this study was conducted to evaluate the influence of four SNPs in ED-associated genes on the prognosis of CC patients.

Regarding the incidence of VTE, among the evaluated SNPs, only *NOS3* rs1799983 (894G > T) demonstrated a significant association with the occurrence of venous thrombotic events. This missense polymorphism is characterised by a guanine (G) to thymine (T) substitution in exon 7 of the *NOS3* gene [22]. This alteration is known to reduce the binding of NOS3 to caveolin-1, thereby lowering its availability in endothelial cells and potentially resulting in a substantial decrease in NOS3 enzyme activity and reduced NO production [23]. Accomplishing the assignment as the main EDRF, NO functions as a vasodilator to maintain endothelial health and exhibits platelet inhibitory properties, thereby regulating vascular health and general physiological haemostasis. A decrease in NO bioavailability is connected with an impaired endothelial function and the subsequent creation of a prothrombotic and proinflammatory environment. This, in turn, has been shown to contribute to both VTE susceptibility and the acceleration of tumour progression [15]. As the SNP T allele is associated with reduced NO levels it was surprising that, in the present study, patients carrying the GG phenotype exhibited a three-fold increased risk of VTE compared to T allele carriers (OR = 3.0; χ^2^ test, *p* = 0.023) However, data specifically addressing the association between *NOS3* rs1799983 and cancer-associated VTE are limited, and findings on its involvement in cancer development are inconsistent, thereby questioning the utility of this SNP as a biomarker [23–26].

Importantly, the genotype distribution of *NOS3* rs1799983 in this study shows a significant deviation from HWE, with fewer heterozygotes (GT) and an excess of both homozygous genotypes (GG and TT). Several factors could contribute to this observation, including chance, population stratification, or technical issues. Also, while speculative, this deviation may reflect selective pressure within the CC population, namely, survival-related natural selection within this cancer cohort may influence the genotype frequencies, as patients with certain genotypes may have differing survival probabilities. Another hypothesis could be that heterozygous carriers are relatively protected against the development of CC, resulting in their underrepresentation in our cohort. Accordingly, the results concerning *NOS3* rs1799983 should be interpreted with caution, as this deviation could overestimate the impact of the GG phenotype. Future research should focus on analysing this relationship in a larger, multi-ethnic cohort to determine whether the mechanistic role of this SNP supports its usage as a predictive biomarker of cancer-associated thrombosis.

Focusing on patient survival, the only SNP found to be a significant predictor of patient death was *NOS3* rs2070744 (-786T > C). This variant is located in the promoter region, modulating *NOS3* expression. The substitution of a T with a cytosine (C) is associated with reduced *NOS3* transcription, which results in lower NO production, potentially leading to an increased risk of cardiovascular disease and cancer progression [22, 27]. In this study, while no direct association with VTE status was observed, *NOS3* rs2070744 was found to be a predictor of CC patient mortality in the subgroup of young women. Specifically, patients carrying the SNP CC genotype (linked to lower NO production) exhibited an almost two-fold increase in 10-year OS compared to T allele carriers, underscoring the potential prognostic relevance of *NOS3* rs2070744 (CC vs. CT/TT; HR = 1.90; log-rank test, *p* = 0.025). These findings are consistent with the existing literature, suggesting a protective role of the T allele in different cancer types, likely due to its association with higher circulating NO levels and the resulting protective effect on vascular health [13, 28–30]. In other words, the poor prognosis linked to the CC genotype, regardless of VTE, can be attributed to the fact that diminished NO levels lead to an inhibition of apoptosis, and increased tumour proliferation, invasion, angiogenesis, and metastasis [15, 28].

On the other hand, no effect of *NOS3* rs2070744 on 10-year OS could be observed in post-menopausal women (> 49 years), suggesting that age and environmental factors may modulate the impact of the SNP. A study on Taiwanese women found that the effect of *NOS3* polymorphisms on breast cancer risk varies significantly according to the menopausal status [26]. The onset of menopause is characterised by a decrease in oestrogen production, a sex hormone that is known to upregulate NOS3 production in endothelial cells [31]. The higher oestrogen levels in premenopausal women enhance NO synthesis and vascular protection, potentially leading to a stronger influence of the rs2070744 CC genotype. In postmenopausal women, who produce lower amounts of oestrogen, the effect of the estrogen-NOS3-NO axis may be diminished, leading to a reduced influence of the rs2070744 variant. Furthermore, environmental factors such as smoking, UV radiation and medical treatments, in addition to the accumulation of free radicals, immune dysregulation and metabolic changes, have the potential to override the effect of the genetic predisposition in this age group, thereby explaining the absence of association between rs2070744 and OS in postmenopausal women [26, 28].

Since preliminary data indicated a potential clinical relevance of *NOS3* rs2070744 in younger CC patients, and this was supported by biological plausibility, the impact of the SNP was subsequently evaluated using a multivariable Cox regression analysis. According to the proposed prognostic models, the incorporation of VTE status in addition to FIGO stage (Model 1) resulted in improved performance in Model 2 (c-index, 0.638 vs. 0.595, respectively). As previously mentioned, VTE negatively impacts cancer patient prognosis due to the disease itself and the close interaction between tumorigenesis and venous thrombogenesis [8]. The transition to Model 3 reveals an intriguing finding. The incorporation of *NOS3* rs2070744, along with FIGO stage, resulted in even higher predictive accuracy (c-index = 0.647), suggesting that this polymorphism may represent a more substantial prognostic factor than VTE status. Model 4, incorporating FIGO stage, VTE status and the SNP, achieved the highest prognostic precision (c-index = 0.685). The 15% relative increase in predictability compared to Model 1 (FIGO stage alone) indicates that *NOS3* rs2070744 could meaningfully contribute to risk stratification and personalised prognostication in younger CC patients. Moreover, these findings highlight the potential of integrating genetic components such as *NOS3* rs2070744 into conventional risk stratification tools, considering that genotyping provides a precise and cost-effective method for identifying genetic risk factors that influence disease development and therapeutic responses [28, 29]. Despite the absence of a direct association between *NOS3* rs2070744 and VTE in the present study, prior research conducted by this group indicated that lower *NOS3* expression is significantly associated with an elevated thrombotic risk [13]. Given the correlation between the variant allele and a decrease in NO levels, the implementation of targeted prophylactic anticoagulation in this subgroup could mitigate potential complications without exposing the broader patient population to unnecessary side effects. However, future studies are needed to explore the underlying mechanisms and confirm the functional role of the SNP. Furthermore, in other conditions in which NO fulfils a regulatory role, such as in cardiovascular diseases in general or in metabolic disorders, genotyping the *NOS3* polymorphism could be clinically relevant. The *NOS3* polymorphism has been associated with variable responses to statins and antihypertensive agents, supporting its utility in personalised medicine [15, 32].

No significant association concerning *VWF* rs1063856 (789T > C) and *SELP* rs6136 (715T > G) was identified in this study. Briefly, the missense variant *VWF* rs1063856 (789T > C) affects the activity of von Willebrand factor (vWF), a glycoprotein that plays a pivotal role in blood clotting, maintaining vascular integrity and inflammation. Specifically, the C allele of the SNP is associated with increased plasma vWF levels [33, 34]. Tumour cells can induce endothelial cells to release vWF [5]. Mechanically, increased levels of vWF result in platelet adhesion and activation, consequently leading to thrombotic events [35]. Additionally, vWF overexpression drives cancer aggressiveness and is associated with more advanced stages of the disease [5, 36]. To the best of our knowledge, this is the first study specifically addressing the association between *vWF* rs1063856 and CC.

Regarding the variant *SELP* rs6136 (715T > G), it is a missense SNP located in exon 13 of the *SELP* gene, which encodes a protein with the same name [37]. The G allele is known to lead to a decrease in P-Selectin (SELP) levels. This cell adhesion molecule facilitates leukocyte recruitment and platelet activation, thereby regulating several immunological processes [38]. Activated platelets and endothelial cells express SELP on their surface, which enables the binding of tumour cells, leading to cell aggregation and promoting blood clotting [39]. Consequently, increased SELP levels can promote tumour metastasis and VTE development [38, 40]. Although SELP has been proposed as a prognostic biomarker and a potential therapeutic target to decrease thrombotic events, existing literature presents conflicting evidence regarding the pathogenicity of *SELP* rs6136 [38]. The inconsistent results may be partially explained by ethnic heterogeneity and distinct exposure to additional risk factors [41]. In the present study, the absence of an association can be attributed to the extremely low minor allele frequency (MAF) of 0.8%, a phenomenon already described in the literature [42]. Specifically, only three patients in our cohort were carriers of the GG genotype, which could have limited the statistical power and thus hindered the detection of more subtle associations. For both SNPs, further research in larger cohorts is required to provide better insights into their role in VTE development and CC patient outcomes.

In this study, the prognostic value of CC histology was evaluated, in addition to patient age, FIGO stage and VTE status, to identify confounding factors. There are considerable discrepancies in the literature regarding the prognostic significance of CC histological subtypes, with some studies suggesting that AC is associated with worse survival outcomes, while others argue that histology plays only a secondary role compared to tumour stage and treatment response [43, 44]. In this study, no prognostic role of CC histology was observed, which is consistent with one of our previous studies [17]. Furthermore, no association was identified between the analysed SNP genotypes and histological subtypes, suggesting that the evaluated genetic variations do not directly drive subtype differentiation. Future research incorporating transcriptomic analyses and tumour microenvironment profiling may assist in the identification of additional genetic signatures that contribute to subtype-specific disease progression.

This study presents certain limitations that may have influenced the detection of significant associations. VTE is a multifactorial condition influenced by a wide range of determinants, including patient-, cancer-, and treatment-specific factors [45]. In addition to cancer, contributory factors include haematological diseases, prior thrombotic events, pregnancy and breastfeeding. Furthermore, the pre-diagnostic utilisation of pharmaceuticals, including oral contraceptives, anticoagulants, and antiplatelet agents, can influence thrombotic susceptibility [45, 46]. Unfortunately, the retrospective nature of this study prevented a more complete characterisation of the study cohort. Thus, residual confounding cannot be fully excluded, and the generalisability of our findings should be interpreted with appropriate caution. Similarly, the analysis of VTE-associated mortality was precluded by the unavailability of cause-specific mortality data. A further significant limitation was the absence of routine VTE screening, which likely resulted in an underestimation of the true incidence of VTE in the investigated cohort. Notably, asymptomatic events account for the majority of cancer-related VTE, negatively impacting patient prognosis [47]. Additionally, patients who experience asymptomatic events are more likely to develop recurrent events, further emphasising the importance of early detection and management [48]. Finally, the relatively small cohort size may have reduced statistical power, particularly in analyses of SNPs with a low MAF. These constraints reflect broader challenges in clinical practice.

On the positive side, this study highlights the potential significance of the ED-associated genetic marker *NOS3* rs2070744 in the prognosis of CC patients, advocating its integration in prognostic models to improve predictive accuracy. Validation studies should prioritise prospective designs with comprehensive data collection, active VTE surveillance, and larger, more diverse cohorts, with appropriate stratification by hormonal and environmental factors. Additionally, studies should incorporate haplotype analyses and explore epigenetic and environmental modifiers to clarify the role of this SNP in CC. Taken together, our results offer a promising foundation for future translational research and highlight the potential of *NOS3* rs2070744 as a valuable tool in personalised cancer treatment.

## Data Availability

The data presented in this study are available on request from the corresponding author.

## References

[1] Costa MT, Neto BV, Brito J et al (2025) Venous Thrombogenesis and Cervical Cancer: Plasma MicroRNAs as Prognostic Indicators of Tumor Behavior. 1–24 10.3390/ijms26199796 PMC12524682 41097060

[2] Sung H, Ferlay J, Siegel RL et al (2021) Global cancer statistics 2020: GLOBOCAN estimates of incidence and mortality worldwide for 36 cancers in 185 countries. CA Cancer J Clin 71:209–249. 10.3322/caac.21660 33538338 10.3322/caac.21660

[3] World Health Organisation (2022) Updated Appendix 3 of the WHO Global NCD Action Plan 2013–2030. https://cdn.who.int/media/docs/default-source/ncds/mnd/2022-app3-technical-annex-v26jan2023.pdf. Accessed 6 Aug 2025

[4] American Cancer Society (2025) Cervical cancer early detection, diagnosis, and staging

[5] Tatsumi K (2024) The pathogenesis of cancer-associated thrombosis. Int J Hematol 119:495–504. 10.1007/s12185-024-03735-x 38421488 10.1007/s12185-024-03735-x

[6] Nussinov R, Yavuz BR, Jang H (2025) Molecular principles underlying aggressive cancers. Sig Transduct Target Ther 10:1–27. 10.1038/s41392-025-02129-7 10.1038/s41392-025-02129-7 PMC11830828 39956859

[7] Khorana AA, Ahrendt SA, Ryan CK et al (2007) Tissue factor expression, angiogenesis, and thrombosis in pancreatic cancer. Clin Cancer Res 13:2870–2875. 10.1158/1078-0432.CCR-06-2351 17504985 10.1158/1078-0432.CCR-06-2351

[8] Mukai M, Oka T (2018) Mechanism and management of cancer-associated thrombosis. J Cardiol 72:89–93. 10.1016/j.jjcc.2018.02.011 29588087 10.1016/j.jjcc.2018.02.011

[9] Temraz S, Moukalled N, Gerotziafas GT et al (2021) Association between radiotherapy and risk of cancer associated venous thromboembolism: a sub-analysis of the COMPASS—CAT study. Cancers (Basel) 13:1–10. 10.3390/cancers13051033 10.3390/cancers13051033 PMC7957620 33801174

[10] Connors JM, Sussman TA, Dryg ID et al (2023) Risks for venous thromboembolism with immune checkpoint inhibitor therapy. Blood 142:2640–2642. 10.1182/blood-2023-187867

[11] Blann AD, Lip GYH (2006) Venous thromboembolism. BMJ 332:215–219 16439400 10.1136/bmj.332.7535.215 PMC1352055

[12] de Melo IG, Tavares V, Pereira D, Medeiros R (2024) Contribution of endothelial dysfunction to cancer susceptibility and progression: a comprehensive narrative review on the genetic risk component. Curr Issues Mol Biol 46:4845–4873. 10.3390/cimb46050292 38785560 10.3390/cimb46050292 PMC11120512

[13] de Melo IG, Tavares V, Savva-Bordalo J et al (2024) Endothelial dysfunction markers in ovarian cancer: VTE risk and tumour prognostic outcomes. Life 14:1–17. 10.3390/life14121630 10.3390/life14121630 PMC11678387 39768338

[14] Fernandes CJ, Morinaga LTK, Alves JL et al (2019) Cancer-associated thrombosis: the when, how and why. Eur Respir Rev 28:1–11. 10.1183/16000617.0119-2018 10.1183/16000617.0119-2018 PMC9488553 30918022

[15] Oliveira-Paula GH, Lacchini R, Tanus-Santos JE (2016) Endothelial nitric oxide synthase: from biochemistry and gene structure to clinical implications of NOS3 polymorphisms. Gene 575:584–599. 10.1016/j.gene.2015.09.061 26428312 10.1016/j.gene.2015.09.061 PMC6728140

[16] Assis J, Pinto R, Freitas-silva M (2023) Venous thromboembolism-related genetic determinant F11 rs4253417 is a potential prognostic factor in ischaemic stroke. 70. 10.1016/j.mcp.2023.101917 10.1016/j.mcp.2023.101917 37364690

[17] Neto BV, Tavares V, da Silva JB et al (2023) Thrombogenesis-associated genetic determinants as predictors of thromboembolism and prognosis in cervical cancer. Sci Rep 13:1–15. 10.1038/s41598-023-36161-w 37308506 10.1038/s41598-023-36161-w PMC10260924

[18] Neto BV, Tavares V, da Silva JB et al (2024) Haemostatic gene variations in cervical cancer-associated venous thrombosis: considerations for clinical strategies. J Thromb Thrombolysis 57:815–827. 10.1007/s11239-024-02983-2 38643313 10.1007/s11239-024-02983-2

[19] Matsuo K, Moeini A, Machida H et al (2016) Significance of venous thromboembolism in women with cervical cancer. Gynecol Oncol 142:405–412. 10.1016/j.ygyno.2016.06.012 27350404 10.1016/j.ygyno.2016.06.012 PMC7643406

[20] Barbera L, Thomas G (2008) Venous thromboembolism in cervical cancer. Lancet Oncol 9:54–60. 10.1016/S1470-2045(07)70409-6 18177817 10.1016/S1470-2045(07)70409-6

[21] Wun T, Law L, Harvey D et al (2003) Increased incidence of symptomatic venous thrombosis in patients with cervical carcinoma treated with concurrent chemotherapy, radiation, and erythropoietin. Cancer 98:1514–1520. 10.1002/cncr.11700 14508840 10.1002/cncr.11700

[22] Lacchini R, Silva PS, Tanus-Santos JE (2010) A pharmacogenetics-based approach to reduce cardiovascular mortality with the prophylactic Sse of Statins. Basic Clin Pharmacol Toxicol 106:357–361. 10.1111/j.1742-7843.2010.00551.x 20210789 10.1111/j.1742-7843.2010.00551.x

[23] Tiucă RA, Pop RM, Tiucă OM et al (2025) NOS3 gene polymorphisms (rs2070744 and rs1799983) and differentiated thyroid cancer: investigating associations with clinical outcomes. Int J Mol Sci 26:1–15. 10.3390/ijms26020759 10.3390/ijms26020759 PMC11765836 39859471

[24] Aouf S, Laribi A, Gabbouj S et al (2019) Contribution of nitric oxide synthase 3 genetic variants to nasopharyngeal carcinoma risk and progression in a Tunisian population. Eur Arch Oto-Rhino-Laryngology 276:1231–1239. 10.1007/s00405-019-05333-8 10.1007/s00405-019-05333-8 30758659

[25] Gao X, Wang J, Wang W et al (2015) ENOS genetic polymorphisms and cancer risk: A meta-analysis and a case-control study of breast cancer. Med (Baltim) 94:1–10. 10.1097/MD.0000000000000972 10.1097/MD.0000000000000972 PMC4504617 26131841

[26] Chen CH, Wu SH, Tseng YM et al (2018) Distinct role of endothelial nitric oxide synthase gene polymorphisms from menopausal status in the patients with sporadic breast cancer in Taiwan. Nitric Oxide 72:1–6. 10.1016/j.niox.2017.10.009 29102546 10.1016/j.niox.2017.10.009

[27] Miyamoto Y, Saito Y, Nakayama M et al (2000) Replication protein A1 reduces transcription of the endothelial nitric oxide synthase gene containing a -786T→C mutation associated with coronary spastic angina. Hum Mol Genet 9:2629–2637. 10.1093/hmg/9.18.2629 11063722 10.1093/hmg/9.18.2629

[28] Devulapalli K, Bhayal AC, Porika SK et al (2015) Association of endothelial nitric oxide synthase gene T-786 C promoter polymorphism with gastric cancer. World J Gastrointest Oncol 7:87–94. 10.4251/wjgo.v7.i7.87 26191352 10.4251/wjgo.v7.i7.87 PMC4501928

[29] Sydorchuk AR, Sydorchuk LP, Gutnitska AF et al (2022) Endothelium function biomarkers and carotid intima-media thickness changes in relation to NOS3 (rs2070744) and GNB3 (rs5443) genes polymorphism in the essential arterial hypertension. Endocr Regul 56:104–114. 10.2478/enr-2022-0012 35489051 10.2478/enr-2022-0012

[30] Lu J, Wei Q, Bondy ML et al (2006) Promoter polymorphism (-786T > C) in the endothelial nitric oxide synthase gene is associated with risk of sporadic breast cancer in non-Hispanic white women age younger than 55 years. Cancer 107:2245–2253. 10.1002/cncr.22269 17063466 10.1002/cncr.22269

[31] Chambliss KL, Shaul PW (2002) Estrogen modulation of endothelial nitric oxide synthase. Endocr Rev 23:665–686. 10.1210/er.2001-0045 12372846 10.1210/er.2001-0045

[32] Silva PS, Fontana V, Luizon MR et al (2013) ENOS and BDKRB2 genotypes affect the antihypertensive responses to Enalapril. Eur J Clin Pharmacol 69:167–177. 10.1007/s00228-012-1326-2 22706620 10.1007/s00228-012-1326-2

[33] Mufti AH, Ogiwara K, Swystun LL et al (2018) The common VWF single nucleotide variants c.2365A.G and c.2385T.C modify VWF biosynthesis and clearance. Blood Adv 2:1585–1594. 10.1182/bloodadvances.2017011643 29980574 10.1182/bloodadvances.2017011643 PMC6039659

[34] Ruggeri ZM (2003) Von Willebrand factor, platelets and endothelial cell interactions. J Thromb Haemost 1:1335–1342. 10.1046/j.1538-7836.2003.00260.x 12871266 10.1046/j.1538-7836.2003.00260.x

[35] Karampinis I, Nowak K, Koett J et al (2023) Von Willebrand factor in the plasma and in the tumor tissue predicts cancer-associated thrombosis and mortality. Haematologica 108:261–266. 10.3324/haematol.2022.281315 36134455 10.3324/haematol.2022.281315 PMC9827172

[36] Wang WS, Lin JK, Lin TC et al (2005) Plasma von Willebrand factor level as a prognostic indicator of patients with metastatic colorectal carcinoma. World J Gastroenterol 11:2166–2170. 10.3748/wjg.v11.i14.2166 15810086 10.3748/wjg.v11.i14.2166 PMC4305789

[37] Marteau JB, Lambert D, Herbeth B et al (2008) P-selectin polymorphisms’influences on P-selectin serum concentrations and on their Familial correlation: the STANISLAS family study. J Thromb Haemost 6:920–927. 10.1111/j.1538-7836.2008.02952.x 18363816 10.1111/j.1538-7836.2008.02952.x

[38] Yeini E, Satchi-Fainaro R (2022) The role of P-selectin in cancer-associated thrombosis and beyond. Thromb Res 213:S22–S28. 10.1016/j.thromres.2021.12.027 36210556 10.1016/j.thromres.2021.12.027

[39] Razak NBA, Jones G, Bhandari M et al (2018) Cancer-associated thrombosis: an overview of mechanisms, risk factors, and treatment. Cancers (Basel) 10:1–21. 10.3390/cancers10100380 10.3390/cancers10100380 PMC6209883 30314362

[40] Coupland LA, Chong BH, Parish CR (2012) Platelets and P-selectin control tumor cell metastasis in an organ-specific manner and independently of NK cells. Cancer Res 72:4662–4671. 10.1158/0008-5472.CAN-11-4010 22836751 10.1158/0008-5472.CAN-11-4010

[41] Zhang X, Zhang C, Ma Z, Zhang Y (2023) Soluble P-selectin level in patients with cancer-associated venous and artery thromboembolism: a systematic review and meta-analysis. Arch Med Sci 19:274–282. 10.5114/aoms/159039 36817657 10.5114/aoms/159039 PMC9897104

[42] Kaur R, Singh J, Kapoor R, Kaur M (2019) Association of SELP polymorphisms with soluble P-Selectin levels and vascular risk in patients with type 2 diabetes mellitus: A Case–Control study. Biochem Genet 57:73–97. 10.1007/s10528-018-9881-6 30047017 10.1007/s10528-018-9881-6

[43] Gallardo-Alvarado L, Cantú-de León D, Ramirez-Morales R et al (2022) Tumor histology is an independent prognostic factor in locally advanced cervical carcinoma: A retrospective study. BMC Cancer 22:1–8. 10.1186/s12885-022-09506-3 35418030 10.1186/s12885-022-09506-3 PMC9006627

[44] Hu K, Wang W, Liu X et al (2018) Comparison of treatment outcomes between squamous cell carcinoma and adenocarcinoma of cervix after definitive radiotherapy or concurrent chemoradiotherapy. Radiat Oncol 13:1–7. 10.1186/s13014-018-1197-5 30558636 10.1186/s13014-018-1197-5 PMC6296025

[45] Tavares V, Pinto R, Assis J et al (2020) Venous thromboembolism GWAS reported genetic makeup and the hallmarks of cancer: linkage to ovarian tumour behaviour. Biochim Biophys Acta - Rev Cancer 1873:1–13. 10.1016/j.bbcan.2019.188331 10.1016/j.bbcan.2019.188331 31689458

[46] Heit JA, Spencer FA, White RH (2016) The epidemiology of venous thromboembolism. J Thromb Thrombolysis 41:3–14. 10.1007/s11239-015-1311-6 26780736 10.1007/s11239-015-1311-6 PMC4715842

[47] Dentali F, Ageno W, Pierfranceschi MG et al (2011) Prognostic relevance of an asymptomatic venous thromboembolism in patients with cancer. J Thromb Haemost 9:1081–1083. 10.1111/j.1538-7836.2011.04259.x 21410640 10.1111/j.1538-7836.2011.04259.x

[48] O’Connell CL, Ghalichi M, Boyle S et al (2008) Unsuspected pulmonary emboli identified on routine cancer staging MDCT scans: impact on cancer survival. Blood 112:1–3. 10.1182/blood.v112.11.3818.381818574030

